# Progression of renal cell carcinoma is inhibited by genistein and radiation in an orthotopic model

**DOI:** 10.1186/1471-2407-7-4

**Published:** 2007-01-09

**Authors:** Gilda G Hillman, Yu Wang, Mingxin Che, Julian J Raffoul, Mark Yudelev, Omer Kucuk, Fazlul H Sarkar

**Affiliations:** 1 Department of Radiation Oncology, Barbara Ann Karmanos Cancer Institute, Wayne State University School of Medicine, Detroit, MI, 48201, USA; 2 Department of Pathology, Barbara Ann Karmanos Cancer Institute, Wayne State University School of Medicine, Detroit, MI, 48201, USA; 3 Division of Hematology/Oncology, Department of Internal Medicine, Barbara Ann Karmanos Cancer Institute, Wayne State University School of Medicine, Detroit, MI, 48201, USA; 4 Harper University Hospital, Detroit, MI, 48201, USA

## Abstract

**Background:**

We have previously reported the potentiation of radiotherapy by the soy isoflavone genistein for prostate cancer using prostate tumor cells *in vitro *and orthotopic prostate tumor models *in vivo*. However, when genistein was used as single therapy in animal models, it promoted metastasis to regional para-aortic lymph nodes. To clarify whether these intriguing adverse effects of genistein are intrinsic to the orthotopic prostate tumor model, or these results could also be recapitulated in another model, we used the orthotopic metastatic KCI-18 renal cell carcinoma (RCC) model established in our laboratory.

**Methods:**

The KCI-18 RCC cell line was generated from a patient with papillary renal cell carcinoma. Following orthotopic renal implantation of KCI-18 RCC cells and serial *in vivo *kidney passages in nude mice, we have established a reliable and predictable metastatic RCC tumor model. Mice bearing established kidney tumors were treated with genistein combined with kidney tumor irradiation. The effect of the therapy was assessed on the primary tumor and metastases to various organs.

**Results:**

In this experimental model, the karyotype and histological characteristics of the human primary tumor are preserved. Tumor cells metastasize from the primary renal tumor to the lungs, liver and mesentery mimicking the progression of RCC in humans. Treatment of established kidney tumors with genistein demonstrated a tendency to stimulate the growth of the primary kidney tumor and increase the incidence of metastasis to the mesentery lining the bowel. In contrast, when given in conjunction with kidney tumor irradiation, genistein significantly inhibited the growth and progression of established kidney tumors. These findings confirm the potentiation of radiotherapy by genistein in the orthotopic RCC model as previously shown in orthotopic models of prostate cancer.

**Conclusion:**

Our studies in both RCC and prostate tumor models demonstrate that the combination of genistein with primary tumor irradiation is a more effective and safer therapeutic approach as the tumor growth and progression are inhibited both in the primary and metastatic sites.

## Background

Renal cell carcinoma (RCC) incidence has increased in recent years with approximately 38,890 new cases each year in the United States of America [1]. The disease is responsible for an estimated 12,840 deaths each year [1]. This increased RCC incidence may be linked to certain risk factors including smoking, obesity, high protein diets and hypertension [2]. Nearly half of the patients present only with localized disease that can be treated by surgical removal [2-4]. However, one third of the patients also present with metastatic disease and half of the patients treated for localized carcinomas subsequently develop metastatic disease [2-4]. The median survival of patients with metastases is only eight months, with a five-year survival rate of less than 10% [2-4]. Patients with metastatic RCC frequently present with pulmonary metastases that are poorly responsive to conventional treatment including most chemotherapeutic drugs, hormones and radiation therapy [2-5]. The treatment of metastatic disease has been and remains a difficult clinical challenge.

To develop new and alternative therapeutic modalities for metastatic disease and to investigate the metastatic progression and the molecular genetics of RCC, various pre-clinical animal models were established (reviewed in ref 6). Among others, tumor xenograft models established in immunodeficient mice by implantation of RCC cells, isolated from a human tumor specimen, have been valuable to assess responsiveness to therapy [6-9]. To investigate the combination of radiotherapy with other treatment modalities, we have established a xenograft metastatic RCC tumor model by orthotopic renal implantation of a new KCI-18 human RCC cell line in athymic nude mice. The KCI-18 RCC model was used to study, *in vivo*, the responsiveness of human RCC primary tumors and metastases to the soy isoflavone genistein and radiation.

Genistein, the most bioactive isoflavone of soybeans, was extensively used in cancer studies and demonstrated inhibition of tumor cell growth *in vitro *by affecting the cell cycle and inducing apoptosis [10]. We further showed that genistein potentiated radiation-induced tumor cell killing [11,12]. This was demonstrated in various human tumor cell lines including RCC cell lines and in particular the KCI-18 line [12]. Genistein significantly increased tumor cell death when given prior to radiation in KCI-18 cells similar to the effect observed with the human PC-3 prostate carcinoma cell line [12]. *In vivo*, we previously showed that genistein potentiated inhibition of prostate tumor growth by radiation and controlled spontaneous metastasis to regional para-aortic lymph nodes using two different orthotopic metastatic prostate tumor models [13,14]. Paradoxically, we discovered that pure genistein, administered as a single treatment modality, promoted increased metastasis to lymph nodes [13]. This intriguing observation was reproduced in two independent orthotopic prostate tumor models, the human PC-3 xenograft model in nude mice [13] and the mouse RM-9 model syngeneic in C57BL/6 mice [14] raising concerns regarding soy-based clinical trials for cancer patients. The goals of the current study were to investigate whether treatment with genistein alone also promotes metastasis in the KCI-18 orthotopic RCC model, and whether genistein combined with primary tumor irradiation is an effective treatment approach for RCC treatment. We found that genistein treatment demonstrated a tendency to stimulate the growth of the primary kidney tumor and increase the incidence of metastasis to the mesentery lining the bowel. In contrast when given in conjunction with kidney tumor irradiation, genistein significantly inhibited the growth and progression of established kidney tumors.

## Methods

### Establishment of KCI-18 human RCC cell line

The human renal cell carcinoma (RCC) cell line designated KCI-18 (KCI for Karmanos Cancer Institute) was established in our laboratory from a primary renal tumor specimen obtained from a patient with papillary RCC (nuclear grade III/IV) [6]. Sterile tumor specimen was delivered to the laboratory immediately after nephrectomy [15]. Fatty tissue was removed and the specimen was minced with scissors into small pieces and dissociated by enzymatic treatment with 0.4 mg/ml collagenase type IV (Sigma Chemical Co., St. Louis, MO) in Dulbecco's modified Eagle's medium with 4.5 g/l glucose supplemented with 10% fetal bovine serum, 2 mM glutamine, 10 mM HEPES buffer, 100 U/ml penicillin/streptomycin and 50 μg/ml gentamicin (Invitrogen, Carlsbad, CA). The mixture was stirred overnight at room temperature and filtered through a wire mesh to exclude undigested material. The cell suspension was washed twice in Hanks' balanced salt solution (HBSS), then viable tumor cells were separated from dead cells by Ficoll-Hypaque density gradient (Histopaque; Sigma) centrifugation [15]. Viable cells obtained from the gradient interface were counted by trypan blue exclusion and cultures of tumor cells were initiated at 0.5 × 10^6 ^cells/ml in 25-cm^2 ^flasks [15]. The cultures were incubated at 37°C in a humidified 5% CO_2 _incubator and split using trypsin-EDTA when confluent. Serial *in vitro *passages led to the generation of KCI-18 cell line.

### Orthotopic KCI-18/IK RCC tumor model

**Figure 1:**
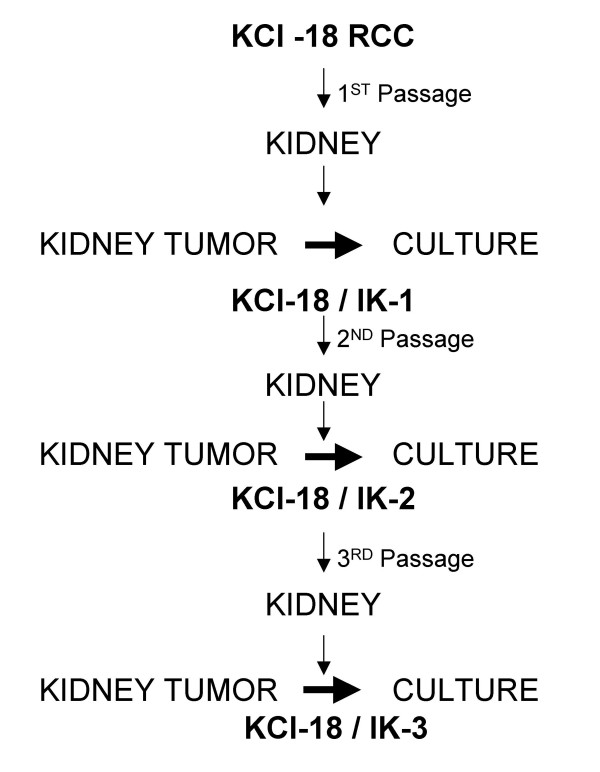
**Generation of KCI-18/IK cell lines**. KCI-18 cells were injected in the kidney of nude mice. Tumor cells were isolated from kidney tumors, expanded in culture and reinjected into mice kidneys. Three serial *in vivo *passages in the kidney resulted in new tumorigenic cell lines KCI-18/IK.

**Figure 2:**
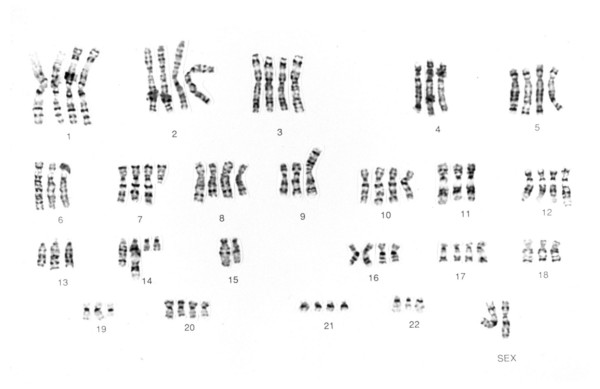
**Chromosomal analysis of KCI-18/IK cell lines**. Karyotype of KCI-18 cells showing near tetraploid chromosome complement and multiple clonal aberrations.

**Figure 3:**
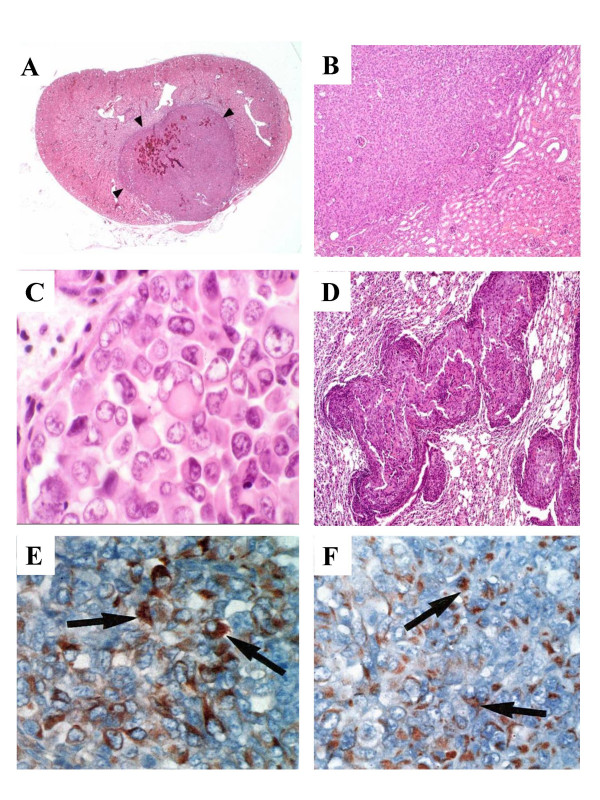
**Histology of KCI-18 kidney tumors and lung metastases**. Following implantation of KCI-18/IK cells in the kidney of nude mice; tumors were resected at different time points and processed for histology. Kidney tumor and lung sections were stained with H&E. Panel A: Development of high-grade carcinoma (arrowheads) with sinusoidal vascular pattern in the kidney (×10). Panel B: Kidney tumor morphology distinct from normal kidney tissue tubular morphology (×25). Panel C: Kidney tumor consisting of cells with large pleomorphic nuclei, prominent nucleoli and abundant eosinophilic cytoplasm (×100). Panel D: Metastatic nodules in lung (×25). Panels E, F: Kidney tumor sections immunostained for cytokeratin (E) and vimentin (F) showing positive cytoplasmic staining for both markers (×50). Magnifications (×-fold) are shown for each picture.

KCI-18 cells were washed twice with HBSS and injected in the right kidney in 5–6 week old female BALB/C *nu/nu *nude immunodeficient mice (Harlan Sprague Dawley Inc., Indianapolis, IN). Mice were anesthetized; the right kidney was exposed through a right flank incision and injected with 1 × 10^6 ^cells in 50 μl HBSS subcapsularly using a 26 gauge needle, then flank wounds were closed with clips [16]. Mice were killed at various time points and kidney tumors were removed in sterile conditions, dissociated in single cell suspensions and cultured *in vitro *to yield a new cell line designated KCI-18/IK1 (IK, intra-kidney implantation). KCI-18/IK1 cells were reinjected in the kidney of nude mice for additional passage in the kidney as shown in Figure 1A. This cycle of *in vivo *kidney passage followed by *in vitro *culture of renal tumors was repeated to produce new KCI-18/IK tumor cell lines (Figure 1). These KCI-18/IK cells were highly tumorigenic, induced metastatic kidney tumors with faster kinetics, and preserved the karyotype of the original KCI-18 cells (Figure 2, 3). Therefore, to assess the effect of therapeutic approaches KCI-18/IK cell lines were used for kidney implantation and a lower concentration of 5 × 10^5 ^cells in 50 μl HBSS was sufficient to produce kidney tumors. Mice were housed and handled under sterile conditions in facilities accredited by the American Association for the Accreditation of Laboratory Animal Care. Mice received Lab Diet # 5021 (Purina Mills, Inc., Richmond, IN), and the animal protocol was approved by the Wayne State University Animal Investigation Committee.

### Irradiation of tumor-bearing organs

**Figure 4:**
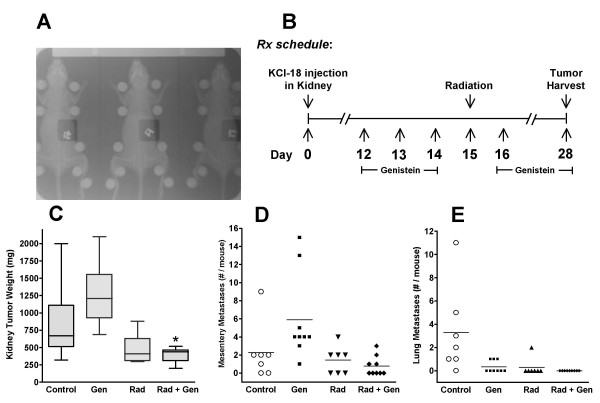
**Treatment of KCI-18 kidney tumors with genistein and radiation**. Panel A: Design of X-ray irradiation of tumor-bearing kidney. Mice in 3 jigs were positioned on an aluminum frame mounted on the X-ray machine. Exposure of the right kidney is confirmed by metallic clips surgically placed in the kidney and visualized in double exposure X ray radiograph while rest of their body was shielded with lead shield positioned above the mice. Panel B: Treatment schedule diagram. On day 12–14 after KCI-18 cell injection in kidney, mice were treated daily with oral genistein at 5 mg/day/mouse. On day 15, established renal tumors were irradiated with 8 Gy photons. One day later, genistein treatment was resumed and given every other day. On day 28, mice were killed and tumors and metastatic tissues were resected. Panel C: Response of primary kidney tumors to radiation and genistein. Weights of the tumor-bearing kidneys and their median weight, from 7–9 mice per treatment group, are reported in boxplot showing the range of data. Panel D: Response of mesentery metastases. Mesentery nodules were prominent along the intestines and enumerated. Each dot represents the number of metastatic nodules per mouse. Panel E: Response of lung metastases. White metastatic nodules were enumerated on lungs insufflated with India ink and bleached as described in Materials and Methods. Each dot represents the number of nodules per mouse. Data from four experimental treatment groups are shown: mice treated with vehicle (Control), genistein only (Gen), radiation only (Rad), genistein combined with radiation (Gen+Rad).

To design selective radiation exposure of the right kidney, metallic clips were surgically placed in the kidney of anesthetized mice. A day later, mice were anesthetized and immobilized in 3 jigs for radiation and positioned on an aluminum frame mounted on the X-ray machine to simultaneously irradiate 3 mice as previously described [13,17]. Shielding was designed based on location of the metallic clips by X ray radiographs (Figure 4A). A 6.4 mm lead shield with 3 cut-outs permitting selective irradiation of the right kidney was positioned above the three mice, as confirmed by double exposure X-ray radiographs (Figure 4A). This allowed for selective irradiation of the right tumor-bearing kidney and shielding the rest of the mouse body, thus establishing the conditions for subsequent experimental radiation treatments (Figure 4B). The radiation dose to the kidney and the scattered dose to areas of the mouse outside of the radiation field were carefully monitored. Photon irradiation was performed with a Siemens Stabilipan X-ray set (Siemens Medical Systems, Inc) operated at 250 kV, 15 mA with 1 mm copper filtration at a distance of 47.5 cm from the target.

### Genistein treatment

Genistein (LKT Laboratories, Toronto, Canada) was dissolved in 0.1 M Na_2_CO_3 _and mixed with sesame seed oil at 2:1 ratio prior to gavage and delivered orally in 0.3 ml at a dose of 5 mg per day per mouse (250 mg/kg body weight per day). This is a feasible and reproducible method allowing us to control the dose administered, as previously described [13,14]. Sesame seed oil was used to facilitate gavage and to avoid irritation of the esophagus by Na_2_CO_3 _[13,14]. Mice from control groups and radiation-only treated groups received a mixture of 0.2 ml 0.1 M Na_2_CO_3 _and 0.1 ml sesame oil.

### Experimental protocol

Before initiating treatment, a few mice were killed to monitor and confirm tumor growth and size in the kidney. In the KCI-18/IK model, small tumors are detectable by day 11 on the kidney. Established tumors were pretreated on days 12–14 for 3 days with oral genistein then on day 15, kidney tumors were irradiated with 8 Gy selected based on radiation dose-titrations. A day later, genistein was resumed and administered every other day for the duration of the experiment (Figure 4B). Seven to nine mice were used per experimental group. Animals were killed on day 28 after tumor cell injection to assess the effect of the treatment on their tumors. At this time point, the tumor burden in untreated control animals was large enough (about 1 × 1 cm in size compared to 0.7 × 0.25 cm normal kidney) to assess and compare the tumor growth in treated animals. Tumor-bearing kidneys were resected and weighed and metastases in the liver, mesentery, and lung were assessed. Tumor nodules in liver or mesentery were visible and were enumerated. Pulmonary metastases were enumerated by insufflating the lungs with a 15% India ink solution using a 21-gauge 1" i.v. catheter. The lungs were then bleached in Fekete's solution allowing detection of white metastatic nodules on a black lung background [18].

### Tissue preparation for histology

At completion of experiments, mice were killed and kidney tumors and metastatic tumor sites including lungs, liver and mesentery tissues were resected and processed for histology studies. The tissues were fixed in 10% buffered formalin, embedded in paraffin and sectioned. Sections were stained with hematoxylin-eosin (H&E) (Figure 2). Tissue sections were immunostained for cytokeratin and vimentin using an avidin-biotin-immunoperoxidase technique [13,19].

### Statistical analysis

Differences in kidney tumor sizes among the various treatment groups were analyzed by two-tailed unpaired t test. Data obtained from all mice treated with genistein combined with radiation were compared to data obtained from control mice, mice treated with genistein only and mice treated with radiation only.

## Results

### Establishment of xenograft metastatic orthotopic renal tumor model

We have established a tumor cell line designated KCI-18 RCC from a primary renal tumor specimen obtained from a patient with papillary renal cell carcinoma (nuclear grade III/IV). Chromosome analysis of KCI-18 RCC cell line (passage 8 *in vitro*) revealed a near tetraploid chromosome complement with multiple clonal aberrations: 75–85, XX, -X, add (1) (p36) x2, +2, +3, +5, +i (5) (q10), +6, +del (7) (q11), +8, der (9;14) (q10;q10), -9, +10, +12, -14, add (15) (q26), +16, +17, +21, +mar, ace [cp6] (Figure 2).

Following serial *in vivo *passages in the kidney of nude mice, KCI-18/IK cell lines were generated (Figure 1) and used to establish a new metastatic RCC experimental tumor model. Cells from the KCI-18/IK lines preserved the karyotype of the original KCI-18 RCC cell line (Figure 2). Following implantation in the kidney, KCI-18/IK cell lines were highly tumorigenic and generated metastatic kidney tumors in 100% of the mice. These tumors grew in the kidney with faster kinetics than the original KCI-18 cell line shortening by 3–4 weeks the development of a metastatic kidney tumor. Primary tumor nodules of about 0.2 cm were detectable on the kidney by day 11 after cell injection and progressively invaded the kidney (Figure 3A, B) reaching sizes as large as 2–2.5 cm by day 40. Metastases were detectable by day 25–30 and observed mostly in the lungs and occasionally in the liver and mesentery. The number and frequency of metastases in the lungs increased with time from 2–5 nodules by day 25–30 to 30–40 nodules by day 40 after cell injection. Mouse survival was about 40–45 days.

The tumor presence in the kidney was histologically confirmed and defined as a high grade carcinoma, highly vascularized with a sinusoidal vascular pattern (Figure 3A, B). Tumor cells were characterized by large pleomorphic nuclei, prominent nucleoli, abundant eosinophilic cytoplasm and large cytoplasmic inclusions (Figure 3C). Tumor nodules in lungs were also clearly detectable by histology (Figure 3D). The cytoplasm of kidney tumor cells stained positively for cytokeratin and vimentin, which are intermediate filaments proteins belonging to the cell cytoskeleton (Figure 3E, F), and known to be co-expressed by various forms of RCC [5,20]. These features of KCI-18/IK kidney tumors resembled the histological characteristics of the original human tumor specimen. These findings demonstrate that we have developed a reproducible renal cell carcinoma tumor model in nude mice in which human tumor cells metastasize from the primary renal tumor to the lungs in a manner that mimics the progression of RCC in human. This model was used to investigate RCC responsiveness to genistein combined with radiation.

### Treatment of kidney tumors with genistein and radiation

Using orthotopic metastatic prostate tumor models, we previously showed that genistein potentiated inhibition of prostate tumor growth by radiation and controlled spontaneous metastasis to regional para-aortic lymph nodes [13]. Paradoxically, we discovered that pure genistein, administered as a single treatment modality, promoted increased metastasis to lymph nodes [13]. This intriguing observation was reproduced in two independent orthotopic prostate tumor models, the human PC-3 xenograft model in nude mice [13] and the mouse RM-9 model syngeneic in C57BL/6 mice (14). To test whether treatment with genistein alone also promotes metastasis in an orthotopic RCC model, but is effective when combined with primary tumor irradiation for RCC treatment, we used the KCI-18 RCC model. Established kidney tumors were pre-treated with 5 mg/day oral genistein for 3 days (day 12–14) followed a day later by tumor-bearing kidney irradiation with 8Gy using conditions described in Materials and Methods (Figure 4A). Then, genistein treatment was resumed every other day at 5 mg/day/mouse and mice were killed on day 28 to assess the effect of therapy on primary tumors and metastatic sites (Figure 4B). This schedule and dose were selected based on our previous studies in the PC-3 prostate tumor model *in vivo *[13] and *in vitro *findings showing that pre-treatment with genistein augments radiation-induced killing both in PC-3 cells and KCI-18 cells [11,12]. In the KCI-18 tumor model, by day 28, large tumor-bearing kidneys in control untreated mice were observed with most of them in the range of 500–1000 mg compared to a weight of 150–200 mg measured in normal kidneys. Compared to control tumors, genistein treatment showed a trend in increasing the growth of kidney tumors resulting in 800–1700 mg weight range (Figure 4C). Radiation slowed the tumor growth (range of 300–650 mg) compared to control tumors but not significantly (p > 0.05) (Figure 4C). However, combination of genistein and radiation caused a significant inhibition of kidney tumor growth (range of 270–400 mg) compared to control tumors and to genistein treated tumors (p < 0.05) (Figure 4C).

Interestingly, following treatment with genistein, we observed a greater number of suspicious nodules in the mesentery lining the intestines that looked like tumor nodules and were histologically confirmed to be tumor nodules (as described in section below, see Figure 6A). Genistein treatment caused an increase in the number and frequency of mesentery metastases compared to untreated control mice and to mice treated with radiation alone or radiation combined with genistein (Figure 4D). In contrast, the number and frequency of lung metastases were limited in mice treated with genistein compared to control mice (Figure 4E). Lung metastases were not observed after radiation and genistein (Figure 4E). Liver metastases were rare in all treatment groups.

### *In situ *analysis of kidney tumors and metastases following genistein and radiation

**Figure 5:**
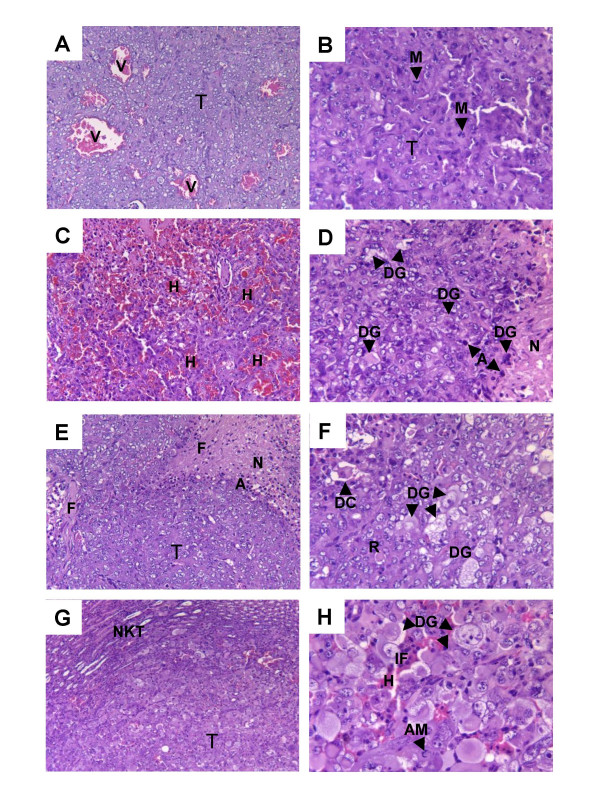
**Histology of KCI-18 kidney tumors treated with genistein and radiation**. Kidney tumors, resected from mice of the experiment described in Figure 4, were processed for histology and tumor sections were stained with H&E. The main findings were labeled on the prints with ***T ***for tumor, ***V ***for vessel, ***M ***for mitosis, ***H ***for hemorrhages, ***DG ***for degenerative, ***N ***for necrosis, ***A ***for apoptosis, ***F ***for fibrosis, ***DC ***for detached cells, ***R ***for rhabdoid cells, ***NKT ***for normal kidney tissue, ***IF ***for inflammatory cells and ***AM ***for abnormal mitosis. Panels A, B: Kidney tumor from control mice showing high-grade and very vascularized carcinoma (A, ×50) with frequent mitosis (B, ×100). Panels C, D: Kidney tumor from mice treated with genistein showing extensive hemorrhages (C, ×50), degenerative changes in tumor cells, apoptotic cells and areas of necrosis (D, ×100). Panels E, F: Irradiated kidney tumor, with areas of tumor destruction, showing fibrosis and apoptotic cells (E, ×50), focal areas with atypical detached rhabdoid cells (F, ×100). Panels G, H: Kidney tumor from mice treated with genistein and radiation, showing smaller residual tumor area adjacent to normal kidney tissue (G, ×25). The residual tumor looked hemorrhagic and consisted of large areas of detached rhabdoid cells, atypical giant cells with large nuclei and inflammatory cells (H, ×100). Lower and higher magnifications (×-fold) are presented to both show wider areas of tumor histology and focus on major findings.

Histological evaluation of kidney tumors, resected on day 28, was performed to determine *in situ *alterations induced by genistein alone compared to genistein and radiation. Large kidney tumors (0.9 × 1 cm) were isolated from control untreated mice and presented histologically as a highly vascularized, high grade carcinoma with some areas of necrosis (Figure 5A). Tumor cells had large pleomorphic nuclei, prominent nucleoli and abundant eosinophilic cytoplasm (Figure 5B). Genistein-treated tumors were large (1 × 1.2 cm) and presented with extensive hemorrhages (Figure 5C). Large areas of necrosis containing apoptotic cells as well as atypical giant tumor cells showing degenerative changes were observed (Figure 5D). Following radiation, tumors were a little smaller (1 × 0.6 cm) than control tumors and contained patchy areas of fibrosis and necrosis infiltrated by apoptotic cells (Figure 5E), as well as hemorrhages but not as prominent as those induced by genistein. Residual viable tumor cells included atypical giant tumor cells or detached rhabdoid cells with large vacuoles (Figure 5F). In contrast to control, or genistein- or radiation- treated tumors in which tumor cells replaced completely normal kidney tissue, tumors treated with genistein combined with radiation were focal covering 40–50% of the kidney and were surrounded by normal kidney tissue consisting of typical tubules and glomeruli (Figure 5G). The residual tumor areas had hemorrhages and consisted mostly of detached rhabdoid cells with large vacuoles and atypical giant cells with pleomorphic nuclei often undergoing degenerative changes (Figure 5H). Some of these tumor-bearing kidneys had small size (0.8 × 0.4) close to that of normal kidney (0.7 × 0.25 cm).

**Figure 6:**
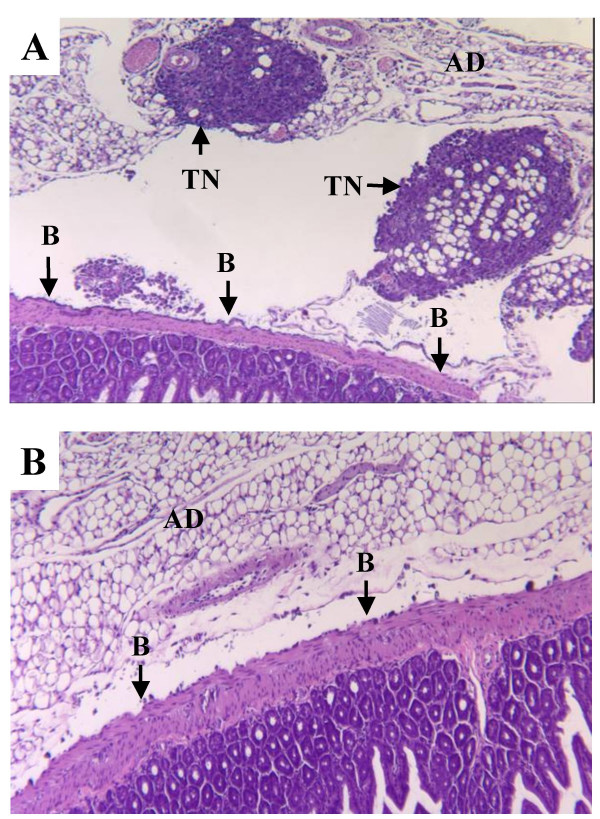
**Histology of mesentery tumor nodules following treatment with genistein**. Sections of the mouse bowel were stained with H&E. Panel A: Section of bowel (***B***) from mice treated with genistein showing tumor nodules (***TN***) in mesentery adipose tissue (***AD***). Panel B: Section of bowel (***B***) from mice treated with genistein and radiation and adipose tissue (***AD***) showing normal morphology of mesentery lining the bowel. Magnifications, ×25.

Following treatment with genistein, we observed a greater number of nodules in the mesentery lining the intestines (Figure 4D). Processing of sections from the bowel tissue, for histology and H&E staining, revealed multiple tumor nodules in mesentery fat tissue that could develop from tumor cell migration through adipose tissue blood vessels (Figure 6A). Staining of this tissue by immunohistochemistry for cytokeratin showed positive staining of tumor cells in the nodules and the morphological characteristics of tumor cells from the primary kidney tumor (data not shown). The bowel looked normal and lymph nodes lining the bowel showed no involvement of tumor. In contrast to genistein treatment alone, following genistein combined with radiation, no tumor nodules were detected in the mesentery lining the bowel in most of the mice (Figure 6B).

## Discussion

We have previously reported the potentiation of radiotherapy by the soy isoflavone genistein. This phenomenon was demonstrated for prostate cancer using prostate tumor cells *in vitro *[11,12] and orthotopic prostate tumor models *in vivo *[13,14]. Genistein combined with primary tumor irradiation caused a greater inhibition of prostate tumors and control of lymph node metastasis than each modality alone. These findings were reproduced in two independent orthotopic tumor models [13,14], however, genistein used as single therapy caused increased metastasis to regional para-aortic lymph nodes [13,14]. This effect was observed with a wide range of genistein doses, from 20 μg/day (Hillman, personal communication) up to 1–5 mg/day [13,14]. To clarify whether these intriguing adverse effects of genistein are intrinsic to the orthotopic prostate tumor model, these studies were repeated in the orthotopic metastatic KCI-18 RCC model established in our laboratory.

The KCI-18 RCC model was established from a cell line generated by culture of cells isolated from a human papillary RCC tumor specimen. Following serial renal implantation of KCI-18 cells in the kidney of nude mice, we confirmed the establishment of a reliable orthotopic RCC experimental model. This model showed the properties of an ideal RCC tumor model including histologically proven carcinoma, predictable growth rate and ability to metastasize similarly to human RCC in a reasonable time frame [6]. The karyotype and histological characteristics of KCI-18 cell line and kidney tumors were comparable to those of the human primary tumor specimen from which the cell line was produced. Like human RCC, these tumors were highly vascularized and stained positively for cytokeratin and vimentin [5,20]. We showed that renal implantation of the KCI-18 cell line in the kidney of nude mice caused the formation of a primary kidney tumor that metastasizes to the lungs, liver and mesentery; mimicking the development and progression of metastatic RCC in human [2,3,21]. Our studies and others demonstrate that orthotopic models of heterotransplanted human RCC cells are representative of human RCC progression and preserve the karyotypic and histological characteristics of the human primary tumor [6-9,20]. These models can be used to study human RCC progression and metastasis and responsiveness of human RCC tumors to treatment *in vivo*.

To investigate the therapeutic effect of genistein and radiation in the KCI-18 model, we first assessed KCI-18 cell responsiveness to these modalities *in vitro*. KCI-18 cells were pre-treated with genistein for 24 hr, then irradiated and assayed in a clonogenic assay in the presence of genistein [12]. KCI-18 RCC cells showed a comparable dose-dependent inhibition of cell growth when treated with genistein alone, but a lower response to radiation compared to PC-3 cells. The combination of 15 μM genistein with 3 Gy radiation, for KCI-18 cells, was more effective at inhibition of colony formation (80%) than genistein alone (45%) or radiation alone (50%) [12]. These data confirm that RCC cell killing by radiation is also enhanced by genistein pre-treatment as demonstrated for PC-3 prostate cancer cells [11,12]. Based on these findings, the treatment of established KCI-18 kidney tumors with genistein alone or combined with radiation was tested.

In the KCI-18 RCC model, treatment of kidney tumor-bearing mice with genistein alone demonstrated a tendency to augment the growth of kidney tumors compared to control mice whereas radiation slowed the growth of kidney tumors. In contrast, genistein given in conjunction with tumor irradiation caused a significant inhibition in the growth of kidney tumors. The anti-tumor activity mediated by genistein and radiation on kidney tumors correlates with the levels of genistein measured in the mice blood. We have previously reported that mice treated with 5 mg/day genistein reached serum levels of about 30 μM compared to 0.2 μM in control mice without apparent toxicity [13]. These levels are comparable to the 15 μM range measured in human volunteers consuming 50 mg isoflavone consisting of 40 mg of genistein and daidzein and in other studies [22,23].

The effects of genistein combined with radiation observed in KCI-18 kidney tumors *in vivo *are in agreement with our *in vitro *data showing a greater inhibition of KCI-18 cell division by the combined treatment. However, the *in vivo *genistein effect of stimulating kidney tumor growth data does not corroborate the findings of KCI-18 tumor cell inhibition *in vitro *suggesting the influence of tumor microenvironment factors *in vivo*.

Interestingly, when used as a single treatment modality, genistein also showed a trend in increasing the number and frequency of tumor nodules in the mesentery lining the bowel but no increase in lung metastasis was noted. Radiation alone and combined with genistein caused limited mesentery metastasis and no lung metastases in the majority of the mice.

These findings were confirmed by histological analysis of the kidney tumors and tissue sections. Genistein-treated kidney tumors showed histological changes comparable to those observed in PC-3 prostate tumors [13], including necrosis, apoptosis and giant tumor cells. As a result of genistein anti-angiogenic effects [24] and the high vascularity of kidney tumors, extensive hemorrhages were observed in genistein-treated kidney tumors. In contrast to genistein or radiation alone, tumors treated with the combined therapy provided histological evidence for significant inhibition of tumor growth and progression in the kidney, with residual focal abnormal tumor cells covering only 40–50% of the kidney. These findings confirm that genistein combined with radiation inhibit the growth and invasion of established kidney tumors and cause marked aberrations in tumor cells that are reminiscent of those observed in prostate tumors [13]. Atypical giant cells are induced by either genistein or radiation alone but are more prominent in tumors treated with both modalities and represent a slow death due to alterations in cell division at the level of cytokinesis [13,17].

Genistein treatment showed a trend in increasing the number and frequency of metastatic mesentery metastases but not that of lung metastases. The mesentery tumor nodules, located in the fat tissue lining the bowel, could have developed from tumor cell migration through adipose tissue blood vessels due to the high vascularization of the kidney tumor. Combining genistein with tumor irradiation controlled the genistein-induced increase of mesentery metastases.

These data indicate that genistein treatment alone has tendency to stimulate the growth of the primary kidney tumor and to promote metastasis to proximal organs but not to distant organs. These findings in the orthotopic RCC model suggest that the effect of genistein on primary tumors located in different organs may differ, as prostate tumor growth was not increased by genistein treatment in orthotopic prostate cancer models. However, genistein promoted metastasis in both RCC and prostate tumor models.

Previous animal studies have emphasized the role of genistein in the prevention of prostate cancer and mammary tumors [25-30]. In contrast, other studies showed increase in tumor growth by genistein in experimental colon cancer in rats, orthotopic mouse mammary tumors and rat prostate tumors [31-33]. Genistein induced stimulation of kidney tumor growth in our orthotopic KCI-12 RCC is in agreement with these studies. Our study emphasizes the adverse effects of pure genistein when treatment is initiated on established tumors.

Clarification of the mechanism by which genistein causes increased metastasis in proximal organs including mesentery in the kidney tumor model or regional lymph nodes in the prostate tumor model is relevant for the clinical application of soy for cancer prevention and cancer treatment. Whether additional isoflavone compounds from the soybean (e.g., daidzein and glycitein) will protect against increased metastasis mediated by purified genistein is currently under investigation.

## Conclusion

The current study, using an orthotopic metastatic RCC model, reproduced the two main findings observed in orthotopic models of prostate cancer, i.e. potentiation of radiotherapy by genistein and tendency of pure genistein to increase metastasis to proximal organs, when used as a single agent. Our studies in both RCC and prostate tumor models demonstrate that the combination of genistein with primary tumor irradiation is a more effective and safer therapeutic approach as the tumor growth and progression are inhibited both in the primary site and metastatic sites.

## Competing interests

The author(s) declare that they have no competing interests.

## Authors' contributions

G.G.H. designed and supervised the study and prepared the manuscript. Y.W. performed the animal experiments. M.C. is a pathologist who analyzed all histology slides with G.G.H. J.J.R. assisted with histology and data analysis. M.Y. is a medical physicist in charge of calibrating the X-ray machine and shielding of the mice. O.K. and F.H.S. participated in the design of the study. All authors read and approved the manuscript.

## Pre-publication history

The pre-publication history for this paper can be accessed here:
